# Impact of a Three-Month Training Break on Swimming Performance in Athletes with Intellectual Disability

**DOI:** 10.3390/sports12120330

**Published:** 2024-12-02

**Authors:** Glykeria Kyriakidou, George Tsalis, Christina Evaggelinou

**Affiliations:** School of Physical Education and Sport Science at Serres, Aristotle University of Thessaloniki, 54124 Thessaloniki, Greece; glikakiriakidou@hotmail.com (G.K.); evaggeli@phed-sr.auth.gr (C.E.)

**Keywords:** freestyle, detraining, physical fitness, body composition, heart rate, strength

## Abstract

This study aimed to ascertain whether there were any differences in anthropometrics, heart rate, and swimming performance parameters in athletes with intellectual disabilities (ID) before and after a three-month training break. A total of 21 athletes participated in the study, comprising 16 males and 5 females, with a mean age of 28.3 ± 8.7 years. All participants had ID, and six of them had Down syndrome. The study participants were classified as S14 athletes from a local swimming club. All participants had a minimum of four years of swimming experience and attended two to three one-hour sessions per week for eight consecutive months. All athletes completed two trials of 25 m freestyle swimming, one at the end of a training session and the other at the beginning of a new session. The measurements included weight, body mass index (BMI), handgrip strength (HGS), heart rate (pre- and post-trial), performance (T25), stroke count (SC), stroke length (SL), stroke rate, and the SWOLF efficiency index. The results demonstrated statistically significant elevations in weight (80.2 ± 16.1 to 81.7 ± 15.9), BMI (26.8 ± 5.5 to 27.2 ± 5.5), T25 (33.1 ± 17.1 to 35.6 ± 18), SC (19.3 ± 6.1 to 20.7 ± 7.2), and SWOLF (52.4 ± 22.0 to 56.3 ± 25.2) and a reduction in SL (1.39 ± 0.48 to 1.27 ± 0.42). However, no significant differences were observed in the remaining parameters. Significant correlations were found for body weight, BMI, HGS, and SWOLF with T25 throughout the study. It was concluded that individuals with ID experienced a decline in 25 m swimming performance due to technical rather than physiological factors after three months of detraining.

## 1. Introduction

Intellectual disability (ID) is characterized by significant limitations both in intellectual functioning and adaptive behavior, as expressed in terms of conceptual, social, and practical adaptive skills. This disability originates during the developmental period, which is defined operationally as before an individual reaches the age of twenty-two [1]. ID is a developmental disorder, and, since it represents a very heterogeneous group, it is usually clinically assessed with appropriately normed, standardized, and validated tests of intellectual functioning and adaptive behavior, which are individually administered [2,3,4]. The measured IQ is considered an approximation of intellectual functioning, which may or may not necessarily correlate with the level of adaptive functioning [4]. According to multiple epidemiological studies, individuals with an IQ score of 50 or below are classified as having severe intellectual disability, whereas those with an IQ score exceeding 50 are classified as having mild intellectual disability [5]. Down syndrome (DS) is a common chromosomal abnormality that can result in various limitations [6]. Individuals with DS often exhibit lower physical fitness (PF) levels compared to those with ID alone [7]. Reduced physical activity (PA) and age-related factors contribute to lower levels of PF in people with ID [8]. Due to the nature of ID, motor function is often impaired, requiring tailored physical development strategies [9].

People with ID exhibit significantly lower muscular strength (MS) than their typically developing peers. For male athletes, this difference is 9%, while, for female athletes, it is 19%. Furthermore, strength differences of up to 40% have been reported for untrained individuals with ID [7,10]. Improved MS has a positive impact on muscle tone and functionality, enhancing the overall movement quality [11]. The heart rate (HR), which is associated with PA, determines one’s energy expenditure and exercise intensity. People with ID typically have lower cardiovascular endurance compared to those without disabilities [12]. The evaluation of effort and endurance is vital in exercise program design, and the maximum heart rate (HRmax) is a key factor in this assessment. It has been observed that people with ID often have a lower HRmax [12].

Swimming is particularly important for people with ID due to the supportive aquatic environment that facilitates movement, especially for those facing gravity-related limitations [13]. Research on the effects of swimming on ID is limited [14]. People with ID benefit from improved cardiorespiratory and muscular endurance, speed, balance, and agility in the water [15,16]. Marques-Aleixo et al. (2013) found that swimmers with ID showed distinctive differences in swimming performance (SP) and MS [17]. Elite swimmers with ID face challenges in speed, particularly during turns, compared to swimmers without ID [18]. Swimmers with ID often encounter challenges in adopting a strategic approach during competitive events, a factor that can adversely impact their performance outcomes [17].

Detraining (DT) is the partial or complete loss of training adaptations that affect performance, as well as one’s anatomical and physiological levels [19]. This observation aligns with the principle of reversibility, which posits that cessation or a reduction in training intensity can lead to regression in physical conditioning. Specifically, the physiological and anatomical adaptations acquired during the preparatory phase are transient and may diminish or revert if the training stimulus is insufficient or discontinued. Consequently, in instances of illness, injury, or rest following a training period, detraining may reverse prior training adaptations, thereby adversely affecting performance parameters. Therefore, athletes should plan their return to training after rest periods to mitigate any measurable reductions in performance capacity [20]. DT can result in a decrease in PF, affecting both cardiopulmonary endurance and MS. The extent of these effects may vary depending on the individual’s level of training [21].

The existing research on sports for individuals with ID is limited, particularly in the context of competitive swimming [22,23,24]. While previous studies have focused on general physical activities or training programs, there is a notable gap in exploring the specific challenges and adaptations required for this population in competitive swimming. The complexities of assessing performance in water, combined with the need for effective collaboration between athletes with ID and researchers, underline the importance of targeted investigations into this area [21]. Furthermore, the existing literature is lacking in its examination of the physiological and performance-related implications of detraining periods in athletes with ID. Detraining represents a crucial phase that can result in a reduction in physical performance and adaptation, particularly in specialized sports such as swimming. This reinforces the necessity for studies that extend beyond general trends and investigate the complex impacts of detraining on key parameters such as body composition, muscular strength, heart rate, and swimming performance.

The present study addresses this gap in the literature by focusing on a three-month detraining period among swimmers with ID, a population that is often underrepresented in sports science research. In contrast to earlier studies that provide generalized insights, this research offers a detailed examination of how detraining affects swimmers with ID, a group that faces unique physiological and environmental challenges [25]. By addressing these aspects, the present study contributes to a deeper understanding of sports adaptations in individuals with ID and provides information to establish more effective training and recovery protocols tailored to their needs. The purpose of this study was to investigate the impact of a three-month detraining period on the body composition, muscular strength, heart rate, and performance among swimmers with intellectual disabilities.

## 2. Materials and Methods

### 2.1. Participants

The sample size (n) required for this study was determined using G*Power 3.1.9.7 for Windows (G*Power, University of Düsseldorf, Düsseldorf, Germany). A sample size of 24 participants was determined to be necessary to detect significant differences with a 95% probability of rejecting the null hypothesis, assuming a medium to large effect size of 0.7 for one group and two measurements. The study involved 21 swimmers, consisting of 16 males and 5 females, with a mean age of 26.2 years (range: 17.6–44.8 years). All participants had ID, and 6 of them had DS. All participants were designated as S14-class swimmers by the National Federation of Sports for People with Disabilities (EAOM-AmeA), specifically for swimming competitions within Greece [26]. Their swimming experience ranged from 4 to 20 years. Each participant engaged in an eight-month training program comprising two or three one-hour sessions per week.

All participants had been diagnosed by a group of certified scientists working at official public disability certification centers. Disability categorization into severe, moderate, or mild ID and DS was based on the WISC-V GR test. Table 1 illustrates the number of participants based on the outcomes of the assessment. The presence of a specific syndrome, such as DS, was included as a criterion for participant selection. The researchers’ acceptance by the participants to ensure the accurate conductance of the measurements constituted an additional criterion. Individuals with significant disabilities received training in small groups of no more than three. These individuals had been trained together for an extended period, which led to the absence of conflicts or issues.

The participants and their guardians were provided with a comprehensive briefing on the procedure, the objective of the task, and the intended utilization of the resulting data. Subsequently, the participants were requested to read and sign the letter of consent for participation in the study, which had been prepared in accordance with the Declaration of Helsinki, prior to the commencement of the experiment. The study was conducted in accordance with the ethical guidelines set forth by the Local Ethics Research Committee of School of Physical Education and Sport Science at Serres, Aristotle University of Thessaloniki (ERC-010/2023, 2 October 2023). The athletes provided their consent for filming to facilitate the analysis of the results. To ensure anonymity, names were matched with codes.

### 2.2. Measurement Procedure

**Figure 1:**
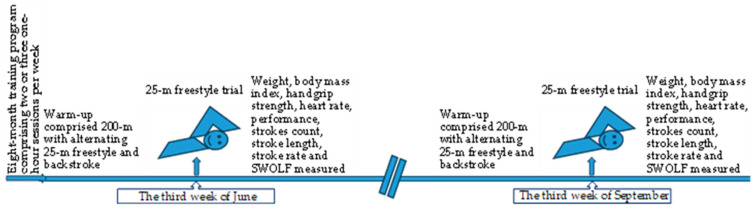
A schematic diagram of the experimental protocol.

Two measurements were taken on two occasions, three months apart, under identical conditions at the National Swimming Pool in Thessaloniki, Greece. The indoor pool was 25 m in length and the temperatures of the water and air were 27 and 25 degrees Celsius, respectively. The measurements were conducted by a doctoral student as part of her thesis, with the assistance of expert colleagues, coaches, and professors. A schematic diagram of the experimental protocol is presented in Figure 1.

### 2.3. Anthropometrics and Performance Parameters

The participant’s date of birth was recorded along with their anthropometric measurements, including their height measured with a Seca 213 stadiometer (Seca GmbH & Co. KG., Hamburg, Germany) and weight measured with a Xiaomi Mi Body Composition Scale 2 Smart (Xiaomi Inc., Pekuno, Beijing, China). The body mass index (BMI) was determined by dividing the body weight, measured in kilograms, by the square of the height, measured in meters.

The maximum force of each hand was measured using the TAKEI 5401 digital hand dynamometer (TAKEI, Niigata-City, Japan) while the athletes were in a standing position. The athletes were required to complete two attempts on each arm with a 15 s interval between attempts, in accordance with the specifications of the Eurofit test [7]. The hand was positioned in accordance with the natural joint angle, and the dynamometer was calibrated to the dimensions of the palm. The optimal performance of each hand was then determined based on these criteria, and, subsequently, the average value of the handgrip force for both hands was calculated and considered.

Following a 30 min recovery period, the subjects engaged in a typical warm-up routine. After 15 min, the time taken by the swimmers to complete a 25 m freestyle at maximum effort was recorded using a CASIO HS 30W handheld stopwatch (Casio Computer Co., Ltd., Tokyo, Japan). The warm-up comprised 200 m with alternating 25 m freestyle and backstroke. During the training period, participants engaged in multiple 25 m freestyle attempts within the program with the objective of enhancing their speed. Additionally, during the training session, the elastic strap of the heart rate monitor was applied to the chest to ascertain the intensity of the participants’ efforts. Following the DT period, the participants undertook one or two training sessions prior to the second trial with the aim of reacquainting themselves with the sensation of swimming in water. The athlete entered the pool, positioning one hand on the start bar of the starting block. Upon the coach’s signal, the swimmer commenced a 25 m freestyle swim. The HR was measured at four time points—pre-trial, immediately post-trial, and at one and two minutes post-trial—using a Polar S410 heart rate monitor (Polar Electro Oy, Kempele, Finland). The participants wore an elastic Polar chest strap with the HR monitor before, during, and for a brief period following the swim. The resting HR was recorded while the participants sat on a chair at the poolside, following a minimum of five minutes of seated rest. At the conclusion of the trial, the HR was measured while the swimmers were in the water and subsequently at one and two minutes post-trial as they returned to the seated position on the poolside chair.

### 2.4. Kinematic Analysis

A digital video camera with a sampling rate of 48 Hz (SJ4000 WiFi digital camera, SJCAM, Shenzhen HongFeng Century Technology Co., Ltd., Shenzhen, China) was placed at a high point on one side and in the middle of the pool [27], twenty meters from the lanes where the swimmers performed their efforts, to record the entire 25 m distance [28]. Before the trials, a 2 m long floating wand was placed in the center of the lane, 2 to 4 m from the wall. The length of the wand in pixels (as measured in screen coordinates) was used to provide an analogy of the screen length to the actual length. This analogy was used to transform the screen length to the actual length for the selected variable. The first 5 m of the 25 m course were marked with a fixed rod outside of the water and excluded from the total distance to ensure the fair measurement of the number of strokes (stroke count—SC20) due to differences in pushing and sliding among the athletes. For each swimmer, the velocity (V20), stroke count (SC20), distance per stroke or stroke length (SL20), and stroke rate (SR20) during the final 20 m of the 25 m freestyle trial were calculated from the recorded data. Additionally, the total time for the 25 m trial (T25) and the total stroke count (SC25) were recorded. The performance index for each athlete, referred to as SWOLF, was calculated [29]. SWOLF, a quantitative measure, originates from the amalgamation of “swimming” and “golf” [30,31]. Like golf, where a lower score signifies improved performance, SWOLF reflects performance in swimming by summing the total strokes taken to complete a specified distance (25 m) and the time required to swim this distance (SWOLF = T25 + SC25). This index provides an indirect measure of performance, offering a valuable means of comparing training sessions within a single individual, rather than between different athletes.

### 2.5. Statistical Analysis

The values are presented as the mean (M) with standard deviation (SD). Shapiro–Wilk’s test was used to check the normal distribution of the data, while Levene’s test was used to test the homogeneity of variance. Paired t-tests were used for all parameters except HR, which was compared using one-way repeated-measures ANOVA. A one-tailed *p*-value was used since the research hypothesis was a deterioration in the parameters tested [32]. The effect size (Cohen’s d, ES) was classified as small (d = 0.20), medium (d = 0.50), and large (d ≥ 0.80). The correlation analysis was conducted using bivariate Pearson’s r analysis between the variables. The statistical analysis was performed using the SPSS software, Version 25.0 (IBM Corp., Armonk, NY, USA). The level of significance was set at *p* = 0.05.

## 3. Results

The mean (M) and standard deviation (SD) are presented, along with the level of significance and Cohen’s d.

### 3.1. Anthropometric Parameters

The final measurement revealed a significant increase of 1.9% in the mean weight and 1.8% in the mean BMI of the participants (*p* = 0.002, d = 2.13 huge; *p* = 0.004, d = 0.74 large, respectively, Table 2).

### 3.2. Heart Rate

**Figure 2:**
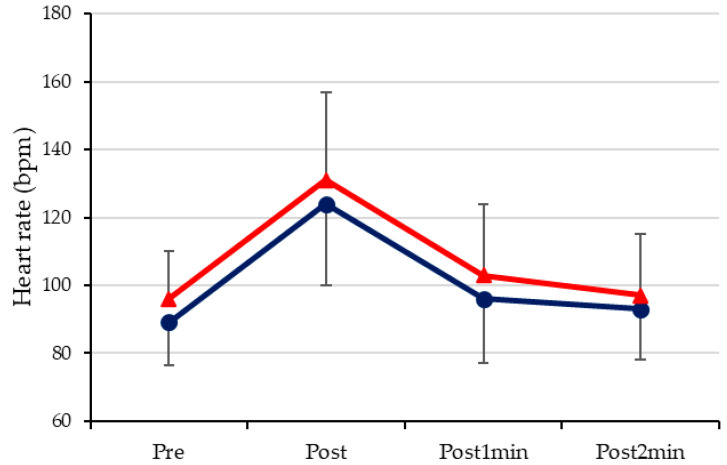
The heart rate measurements taken prior to (Pre), immediately following (Post), one minute after (Post1min), and two minutes after (Post2min) the 25 m freestyle trials are represented in the graph. Heart rate data from the June trials are represented by a blue line, while data from the September trials are indicated by a red line. bpm = beats per minute.

Despite an approximately 6.4% increase in HR across all time points during the September trials, no statistically significant differences were observed in any measurements at any time point (Figure 2). The recorded beats per minute were, on average, 92, 128, 99, and 95 before the trial, immediately after the trial, and at one and two minutes post-trial, respectively.

### 3.3. Performance and Kinematic Parameters

Table 3 shows the performance and kinematic parameters of the participants before and after the summer break. T25 increased significantly by 2.5 s (*p* = 0.002, d = 3.48 huge). In the 20 m swim, the speed was reduced by 0.05 m·s^−1^ (*p* = 0.003, d = 0.08 huge), the SC increase by 1.4 cycles (*p* = 0.003, d = 2.16 huge), and the SL was reduced by 0.12 m (*p* < 0.001, d = 0.13 small), but the SR was remained at around 35.4 cycles·min^−1^ (*p* = 0.491, d = 4.06 huge). The SC at 25 m increased by 1.5 cycles (*p* = 0.003, d = 2.16 huge), and the SWOLF efficiency index worsened from 52.4 to 56.3 points (*p* < 0.001, d = 4.84 huge).

There were no statistically significant differences between the two measures of the handgrip strength, which was approximately 226 Ν (*p* = 0.070, d = 65.50 huge).

No significant correlations were observed between the HR and any of the performance parameters across the total participant cohort during the study. Table 4 presents the correlation analysis results, revealing that an increased body weight was associated with a prolonged T25 in both measurements (r = 0.479, *p* = 0.028; r = 0.489, *p* = 0.024, respectively) and had an inverse relationship with the SR20 (r = −0.562, *p* = 0.008; r = −0.496, *p* = 0.022, respectively). The BMI demonstrated a strong positive correlation with both an elevated T25 and SWOLF (r = 0.731, *p* = 0.000; r = 0.703, *p* = 0.000 and r = 0.684, *p* = 0.001; r = 0.680, *p* = 0.001, respectively) across both measurements, but a negative association with the SC20 (r = −0.537, *p* = 0.012), specifically in September. Additionally, the HGS was negatively correlated with the T25 across both measurements (r = −0.458, *p* = 0.037; r = −0.470, *p* = 0.032, respectively), and, in September, the HGS showed a positive correlation with the SL20 (r = 0.551, *p* = 0.010), while presenting an inverse relationship with both the SC20 and SWOLF (r = −0.475, *p* = 0.029; r = −0.488, *p* = 0.025, respectively).

## 4. Discussion

It is well established that the cessation of training can lead to the reversal of training-induced adaptations, thereby impacting performance parameters. Specifically, detraining has been shown to result in declines in PF, notably in cardiopulmonary endurance and MS, with the extent of these declines depending on the individual’s baseline training level [20,21,25]. While extensive research has examined this phenomenon among individuals without disabilities, the effects on individuals with ID remain largely unexplored. This study aimed to advance the understanding of the impacts of a three-month period of DT on the body composition, strength, HR, and performance in swimmers with ID.

### 4.1. Anthropometric Parameters

The present study examined the impact of DT on anthropometric measurements, specifically weight and BMI. Most participants exhibited an increase in weight and BMI, with a mean increase of 1.9% and 1.8%, respectively, over the course of the three-month DT period (Table 2). At the outset of the study, 50% of the participants exhibited a normal BMI (18.5–24.9 kg/m^2^), 25% were classified as overweight (BMI > 25–29.9 kg/m^2^), and the remaining participants were obese (BMI ≥ 30 kg/m^2^) [33]. However, this status deteriorated following the observation of two individuals transitioning from a state of normal weight to one of overweight, with most participants exhibiting a shift in their BMI category. There is substantial evidence that regular exercise prevents weight gain and an increase in BMI [34]. It is evident that the observed increase in weight and BMI was a consequence of the lack of regular exercise. These effects have been observed in individuals with ID, as well as in the general population [35,36,37]. Although the present study did not record the health status of the participants before and after the DT period, it is established that weight gain and an increase in BMI can have a negative impact on the overall health of people with ID [6,38,39].

### 4.2. Heart Rate

The objective was to measure the HR to determine the intensity of cardiac effort during rest, after exercise, and at 1 and 2 min post-exercise. Additionally, the aim was to ascertain whether there were any changes in HR after DT. The results demonstrated no statistically significant differences in HR at rest or after the test. These findings are consistent with those of Zacca et al. (2019), who also observed that four weeks of DT were insufficient to induce alterations in the myocardial structure [40].

This study revealed that there was an approximately 6.4% increase in HR in September in comparison to June (Figure 2). Coyle et al. (1984) observed a 5% increase in HR in athletes who had undergone aerobic training after a period of 84 days during which they did not engage in any training [41]. This finding suggests that DT may have a negative impact on cardiovascular fitness. The lack of cardiorespiratory training may be due to a slight loss of adaptation [19]. This study found that the highest HR achieved during swimming was approximately 75% of the predicted HRmax, as calculated using the formula Y = 189 − (0.59·age) for subjects with ID or Y = 210 − [(0.56·age) − 15.5] for subjects with DS [12]. The findings indicated that the subjects were unable to reach the requisite exertion levels during the trial. Additionally, the data demonstrated that their HRmax was approximately 15 beats per minute lower than that observed in their peers without ID [8]. Individuals with ID have been shown to exhibit lower HR levels in response to stress stimuli compared to the general population [42]. This results in a plateau that may not be affected by abstinence from exercise.

However, these results suggest that longer or more intensive training programs may be necessary to elicit measurable physiological changes in athletes with ID. Additionally, HR data alone may not fully capture the degrees of training adaptation in this population. The present study did not calculate the heart rate variability (HRV), which is a more sensitive indicator of autonomic nervous system regulation and has shown value in assessing cardiovascular recovery and adaptation. HRV provides insights into the balance between sympathetic and parasympathetic activity, which is critical in understanding training effects and recovery. Esco and Flatt (2014) highlighted the utility of ultra-short-term HRV measurements in monitoring training adaptations, particularly in athletic populations [43]. There is significant autonomic dysfunction in individuals with ID, which manifests as impaired regulation of the autonomic nervous system both at rest and during physical activity, indicating an altered balance between sympathetic and parasympathetic activity [44]. The implications of the HR and HRV indicators are significant for the design of training programs. The identification of HR zones, based on individual responses, can optimize the training intensity. Furthermore, HRV monitoring can help to prevent overtraining and assess the readiness to train, emphasizing the importance of the BMI in understanding how athletes adapt to varying workloads [45].

The average time required to complete the 25 m swim was approximately 35 s in both measurement trials, which may not have been sufficient to produce a significant elevation in HR. However, the highest HR reached approximately 75% of the predicted maximum HR, indicating a relatively modest proportion. Notably, individuals with ID demonstrated an SR considerably lower than that of typical swimmers, averaging approximately 35 cycles per minute in this research, compared to around 49 cycles per minute in López-Plaza et al.’s (2024) study [29]. Interestingly, a similar proportion (~75%) was observed in the SR and HR for individuals with ID relative to competitive swimmers. These results suggest that individuals with ID have a limited capacity for high-intensity swimming, leading to a less substantial increase in HR.

Furthermore, no significant correlations were observed between the HR and any performance parameters across the entire participant cohort in this study.

### 4.3. Performance and Kinematic Parameters

There was a significant decline in performance of 2.5 s after DT, consistently with a decrease in V20 by 0.05 m·s^−1^. Research has shown that swimmers’ performance can be negatively affected by interruptions in training lasting either 4–6 weeks or longer than 10 weeks [40,46]. Głyk et al. (2022) reported a decrease in swimming speed in adult swimmers after 3 months of DT [47]. SC, SL, and SR are important parameters in determining the swimming speed and achieving better times for a given distance [48,49,50]. In this study, the SC20 and SC25 increased significantly by 1.4 SC and the SL20 decreased significantly by 0.12 m between the two measurements. However, the SR20 remained constant at around 35 cycles·min^−1^. The data suggest that the SR was the key factor affecting performance. Changes in SC and SL were significantly related to changes in the swimming speed, resulting in a decrease in SP [24]. The highest performance was achieved in the June measurement, where the participants had a superior SC and SL. There appeared to be no difference in the pattern of SP between the typical group and the population with ID [24]. The SWOLF index demonstrated a statistically significant difference as it increased from a mean of 52.4 to 56.3 points. This indicates a loss of kinetics and physical adaptation from training. While there were very small differences in the individual performance parameters, the effectiveness of the swimming ability declined [51].

The relationship between an athlete’s mental and motor potential can be influenced by the level of brain development. Giagazoglou et al. (2012) suggest that inadequate brain development can restrict an athlete’s mental potential [9]. Marques-Aleixo et al. (2013) found that people with ID may have poor strategic performance in swimming due to their limited cognitive abilities [17]. The present study confirms the relationship between swimming speed parameters and performance, consistent with typical swimmers. A higher SC resulted in a prolonged time. Furthermore, better performance was correlated with a longer SL, higher SR, and a lesser SWOLF index [52]. To minimize the negative effects of detraining, coaches should prioritize the incorporation of technical exercises that are specifically designed to optimize the body position and reduce resistance. The combination of resistance training with targeted feedback has been demonstrated to assist swimmers in restoring their propulsion efficiency [53]. In terms of implementing exercises that focus on the body position and smoothing techniques that enhance propulsion and reduce resistance, it is emphasized that technical feedback during training is important to optimize swimmers’ mechanics. Furthermore, it is recommended that resistance training (e.g., tethered swimming, resistance training) be incorporated. However, it is noteworthy that the latter approach appears to be more applicable and effective in individuals with ID [9]. Furthermore, interval training and aerobic conditioning can enhance the SWOLF index by improving the swimming economy [51].

Athletic performance is a complex function shaped by various physiological, psychological, and cognitive factors. Deficits in intellectual functioning are thought to influence athletic performance, although empirical data are limited. Research has shown that athletes with intellectual disabilities tend to achieve lower performance levels compared to athletes without intellectual disabilities, exhibiting reduced speeds and acceleration in athletics, less efficiency in swimming, and diminished technical proficiency in sports like table tennis [54]. These findings suggest that athletes with intellectual disabilities face unique challenges in achieving peak performance, particularly in sports that require high technical skill and coordination [55]. Cognitive abilities and executive functioning play a critical role in achieving proficiency in sports. Consequently, the performance of athletes with ID is likely to remain influenced by their underlying cognitive impairments, regardless of the intensity or extent of their training efforts [56].

The correlation analysis showed that both body weight and BMI were associated with a prolonged T25, as well as a decline in SWOLF and poor performance on both measures. In a typical population of swimmers, the BMI does not seem to have an impact on performance [51,57]. However, people with ID who have a high BMI are often described as having low levels of cardiorespiratory fitness [16,58] or poor motor skills [59,60].

### 4.4. Muscular Strength

Following detraining (DT), an observed increase in HGS from 215 N to 237 N was noted, although it did not reach statistical significance. This outcome aligns with the findings of Lemmer et al. (2000), which demonstrated that the strength gains resulting from training were comparably retained across both young and older men and women during a 12-week period of detraining [61]. Additionally, in both measurement periods, a correlation was identified between the HGS and T25, consistent with prior research by Garrido et al. (2012) and Zampagni et al. (2006) [62,63].

### 4.5. Limitations

It is essential to acknowledge the limitations of this study. The sample size was relatively small, but it included individuals with intellectual disabilities across a wide range of ages and levels of disability. Furthermore, the relatively limited number of individuals with Down syndrome included in the study, along with the overall sample size of 21 participants, represents a notable limitation. The stratification of the participants by gender and disability status would have resulted in the formation of small subgroup sizes, which would have further reduced the statistical power and robustness of the analysis.

## 5. Conclusions

The findings indicated a statistically significant decline in performance, with the event times increasing by an average of 2.5 s from June to September. This decline was attributed to a reduction in the SC, SL, and SWOLF index, which indicates a decline in swimming efficiency. Athletes with higher SCs demonstrated a reduction in glide, flexibility, and propulsion effectiveness, which is consistent with the principle of reversibility. This principle highlights the loss of training adaptation during periods of inactivity. The positive correlation between grip strength and performance emphasized the significance of muscular strength, while the slight increase in heart rate and physiological changes, including weight and BMI increases, during the detraining period highlighted cardiovascular and health risks. Athletes with ID encountered additional challenges, including the inability to reach optimal heart rate levels. This study underscores the vital importance of consistent physical activity for the maintenance of fitness and the mitigation of detraining effects, particularly in the context of athletes with ID. However, the findings’ generalizability is limited by the small sample size. It would be beneficial for future research to incorporate a more diverse population, including athletes with varying intellectual abilities, ages, and training backgrounds. Additionally, longitudinal studies spanning multiple seasons would be advantageous in exploring the long-term detraining effects. Tailored interventions for individuals with ID are essential to address their unique challenges and support the development of inclusive, evidence-based training protocols.

### Perspectives

The effects of physical activity cessation in individuals with ID, particularly in relation to swimming, have been the subject of limited research. Swimming has been demonstrated to enhance the quality of life of people with ID, while also facilitating their participation in competitive activities and enabling them to achieve their full potential, thereby promoting socialization. In order to enhance the detection of parameter variations, it is recommended that the parameters in question be measured during the training period. Furthermore, future research could consider recruiting participants exhibiting a wider range of swimming styles and distances.

## Figures and Tables

**Table 1:** Disability severity of participants with ID.

Severity of Disability	Male	Female
Mild	5	0
Moderate	9	3
Severe	2	2

**Table 2:** The age, height, weight, and body mass index (BMI) of the participants are presented, along with the changes in the absolute values and percentages before and after the cessation of training. The results are presented as the mean (M) and standard deviation (SD).

	June		September		*p*	d	Change
	M	SD	M	SD			
Age (years)	28.1	8.7	28.4	8.7			
Height (m)	1.74	0.1	1.74	0.1			
Weight (kg)	80.2	16.1	81.7	15.9 *	0.002	2.13	↑ 1.5 kg or 1.9%
ΒΜΙ (kg/m^2^)	26.8	5.5	27.2	5.5 *	0.004	0.74	↑ 0.4 kg/m^2^ or 1.8%

m = meters; kg = kilograms; ΒΜΙ = body mass index; ↑ = increasing, * statistically significant difference between periods.

**Table 3:** Results of performance and kinematic characteristics before and after the summer break. Results are presented as mean (M) and standard deviation (SD).

	June		September		*p*	d
	M	SD	M	SD		
T25 (s)	33.1	17.1	35.6	18.8 *	0.002	3.48
V20 (m·s^−1^)	0.78	0.23	0.73	0.23 *	0.003	0.08
SC20 (cycles)	16.3	6.1	17.7	7.2 *	0.003	2.16
SL20 (m)	1.39	0.48	1.27	0.42 *	<0.001	0.13
SR20 (cycles·min^−1^)	35.4	10.4	35.4	9.2	0.491	4.06
SC25 (cycles)	19.3	6.1	20.7	7.2 *	0.003	2.16
SWOLF	52.4	22.0	56.3	25.2 *	<0.001	4.84
HGS (Ν)	215	71	237	70	0.070	65.50

T25 = 25 m swim time; V20 = 20 m swim velocity; SC20 = number of strokes in a 20 m swim; SL20 = distance per stroke in a 20 m swim; SR20 = stroke rate in 20 m swim; SC25 = number of strokes in a 25 m swim; SWOLF = performance index; HGS = handgrip strength; Ν = newton. * significantly different between periods.

**Table 4:** Notable correlations among the examined parameters.

	June		September	
	Pearson’s r	*p*	Pearson’s r	*p*
WEIGHT—T25	0.479	0.028	0.489	0.024
BMI—T25	0.731	0.000	0.703	0.000
BMI—SWOLF	0.684	0.001	0.680	0.001
HGS—T25	−0.458	0.037	−0.470	0.032
SWOLF—T25	0.981	0,000	0.987	0.000

T25 = 25 m swim time; SR20 = stroke rate in 20 m swim; ΒΜΙ = body mass index; SC20 = number of strokes in a 20 m swim; SWOLF = performance index; HGS = handgrip strength; SL20 = distance per stroke in a 20 m swim.

## Data Availability

The data that support the findings of this study are available upon request to the corresponding author.
